# Metformin exerts an antitumor effect by inhibiting bladder cancer cell migration and growth, and promoting apoptosis through the PI3K/AKT/mTOR pathway

**DOI:** 10.1186/s12894-022-01027-2

**Published:** 2022-05-24

**Authors:** Zhiyong Shen, Dong Xue, Kun Wang, Facai Zhang, Jiaqi Shi, Benzhong Jia, Dan Yang, Qianjin Zhang, Shuai Zhang, Hongyu Jiang, Daiqin Luo, Xueying Li, Quliang Zhong, Junhao Zhang, Zheng Peng, Yu Han, Chongyang Sima, Xiaozhou He, Lin Hao

**Affiliations:** 1 Department of Urology, The Third Affiliated Hospital of Soochow University, No.185, Juqian Street, Tianning District, Changzhou, 213000 Jiangsu Province China; 2 Department of Urology, The Affiliated Cancer Hospital of Guizhou Medical University, Guiyang, Guizhou Province China; 3 Department of Urology/Institute of Urology, West China Hospital, Sichuan University, Chengdu, Sichuan Province China; 4 Department of Urology, The Affiliated Hospital of Guizhou Medical University, Guiyang, Guizhou Province China; 5 Laboratory of the Affiliated Cancer Hospital of Guizhou Medical University, Guiyang, Guizhou Province China; 6 Department of Clinic Research Center, The Affiliated Hospital of Guizhou Medical University, Guiyang, Guizhou Province China; 7 Department of Urology, The Affiliated Suqian First People’s Hospital of Nanjing Medical University, Nanjing, China; 8 Department of Urology, Xuzhou Central Hospital, No. 199 Jiefang Street, Quanshan District, Xuzhou, 221009 Jiangsu China

**Keywords:** Bladder cancer T24 cells, Bladder cancer 5637 cells, Metformin, PI3K/AKT/mTOR signaling pathway

## Abstract

**Background:**

To observe and explore the effect of metformin on the migration and proliferation of bladder cancer T24 and 5637 cells in vitro*.*

**Methods:**

Bladder cancer T24 and 5637 cell lines were cultured in vitro, and were divided into group A (blank control group) and group B (metformin group: 5, 10, 15, and 20 mmol/L); both groups were plated on 6-well plates at the same time. Culture in 24-well plates was used for wound healing assays and in 96-well plates for Transwell migration and invasion, and Cell Counting Kit-8 proliferation experiments. We observed and detected the cell migration and proliferation ability of each group at 48 h, and calculated the cell migration area and survival rate. Flow cytometry was used to detect cell apoptosis in the groups. The apoptosis-related proteins, cleaved-caspase 3, cleaved-PARP, and the PI3K/AKT/mTOR signaling pathway member proteins PI3K, phosphorylated (p)-PI3K, AKT, p-AKT, mTOR, and p-mTOR were detected using western blotting.

**Results:**

After 48 h of treatment with different concentrations of metformin, the cell migration and proliferation capabilities were significantly lower than those in the blank control group. The proliferation and migration abilities of T24 and 5637 cells decreased in a metformin concentration-dependent manner (*P* < 0.05). The apoptosis rate under different concentrations of metformin, as detected by flow cytometry, showed a significantly higher rate in the metformin group than in the control group (*P* < 0.05). Compared with that in the control group, the level of cleaved-caspase 3 and cleaved-PARP protein in the metformin group was increased in each treatment group, and the levels of p-mTOR, p-AKT, and p-PI3K decreased significantly compared with those in the control group (*P* < 0.05).

**Conclusion:**

Metformin inhibited bladder cancer T24 and 5637 cell migration and proliferation, and induced their apoptosis. The mechanism might involve inhibition of the activation of the PI3K/AKT/mTOR signaling pathway.

**Supplementary Information:**

The online version contains supplementary material available at 10.1186/s12894-022-01027-2.

## Background

Bladder cancer is a common malignant tumor of the urinary system [1]. Clinically, approximately 70% of bladder cancer cases are non-muscular invasive bladder cancer (NMIBC) and 30% are muscularly invasive bladder cancer (MIBC) [2, 3]. NMIBC is characterized by a high rate of recurrence and a low rate of mortality. For NMIBC, the preferred surgical treatment method is transurethral electric resection of bladder tumor (TURBT) and routine postoperative infusion of chemotherapy drugs to prevent tumor recurrence and progression; however, active postoperative adjuvant therapy still results in a high recurrence rate. Metformin is a clinical drug used routinely to treat type 2 diabetes. We reviewed the domestic and foreign literature and found that metformin can significantly reduce the incidence of pancreatic cancer, liver cancer, breast cancer, and other malignant tumors [3, 4], in addition to improving the prognosis of patients with type 2 diabetes [5, 6]. These findings suggested that metformin had a potential antitumor effect. Therefore, from September 2019 to May 2021, we observed metformin’s effects on bladder cancer T24 and 5637 cell proliferation and migration, and explored its possible molecular mechanisms. We aimed to provide a theoretical basis for the use of metformin to treat bladder cancer.

## Methods

### Reagents and cell culture

#### Reagents

Human bladder cancer cell lines T24 and 5637 were provided by the cell bank of the Chinese Academy of Sciences in Shanghai. The present study used metformin (Shanghai Beyotime Biotechnology Co., Ltd., Shanghai, China), Roswell Park Memorial Institute (RPMI)-1640 medium (Cellmax Company, Groningen, Netherlands), fetal bovine serum (FBS) (Sigma, St. Louis, MO, USA), phosphate-buffered saline (PBS) (Beijing Solarbio Company, Beijing, China), Cell counting Kit-8 (CCK-8) (Shanghai Beyotime Biotechnology Co., Ltd., Shanghai, China), and an Annexin V-fluorescein isothiocyanate (FITC)/propidium iodide (PI) Apoptosis Assay Kit (BD Biosciences, San Jose, CA, USA). Antibodies recognizing PI3K/Akt/mTOR signaling pathway associated proteins (phosphatidylinositol-4,5-bisphosphate 3-kinase (PI3K), phosphorylated (p)-PI3K, protein kinase B (Akt), p-Akt, mechanistic target of rapamycin (mTOR), p-mTOR), glyceraldehyde-3-phosphate dehydrogenase (GAPDH), and Apoptosis-related protein (cleaved-caspase 3 and cleaved-poly(ADP-ribose) polymerase (PARP)) were all purchased from Affinity Biosciences (Cincinnati, OH, USA). We also used a microplate analyzer (Thermo Scientific, Waltham, MA, USA), a flow cytometer (Bio-Rad, Hercules, CA, USA), and a western blotting imaging system (Bio-Rad).

#### Cell culture

Bladder cancer T24 and 5637 cells were taken out of − 80 °C liquid nitrogen storage and thawed in a beaker for 1 min. The cells were suspended in RPMI-1640 medium containing 10% FBS and placed into an incubator at 37 °C and 5% CO_2_ for culture. After the cell density reached about 90%, the cells were passaged. T24 and 5637 cells in the logarithmic growth phase were used for the following experiments.

### Wound healing assay

We made horizontal lines on the back of the 6-well plate, about every 0.5–1 cm, across the holes. A single cell suspension was prepared by adding 0.25% trypsin to the cells for digestion. The cell plate count method was used to adjust the cell concentration to about 5.0 × 10^5^/mL and the cells were inoculated into a 6-well plate. The control group (A) and Group B (metformin 5, 10, 15, and 20 mmol/L) were placed in an incubator for 24 h until the cells were attached to the wall and a confluent cell layer had developed. Scratches were made in the cell layer using a micropipette tip. The cells were washed with PBS three times to remove the scratched cells. Serum-free RPMI-1640 medium was added vertically and gently at the apex of the plate. Photographs were taken at time zero and after 48 h of culture using an Olympus camera (Tokyo, Japan).

### Transwell assay

A single cell suspension was prepared by adding 0.25% trypsin to the cells for digestion. 500 μL of medium containing 10% FBS was added to the lower chamber of the Transwell apparatus (8 μm in diameter), and 200 μL of cell suspension without serum was added to the upper chamber. After incubation for 48 h, the medium in the upper and lower chambers was removed, and 500 μL of 4% paraformaldehyde was added to the lower chamber to fix the cells for half an hour, after which the paraformaldehyde was removed. The cells that did not penetrate the membrane were wiped off using a cotton swab. Then, 500 μL of 0.1% crystal violet were added to the lower chamber to stain the cells for 15 min. The chamber was washed with PBS until it was completely clear, and the cells were observed and counted under a microscope (Olympus).

### Assay for cell growth

A Cell Counting Kit-8 (CCK-8) assay was used to determine cell proliferation. Logarithmic growth phase cells were digested with 0.25% trypsin, and a single cell suspension was prepared. The cell density was adjusted to about 1 × 10^3^/mL, and 100 μL cells of the suspension were inoculated in each well of a 96-well plate, and cultured in an incubator for 24 h. After the cells adhered to the wall, the medium was discarded and 5, 10, 15, and 20 mmol\L metformin were added and incubated for 48 h, respectively. Five replicates were set for each concentration, and a blank control group was set with the same volume of medium. After the treatment, each well received 10 μL of CCK-8 solution, the 96-well plate was gently knocked to mix, and then incubated for 1.5 h. The absorbance value at 450 nm was measured using an enzyme plate analyzer (Thermo Scientific).

### Assay for colony formation

The proliferation of T24 and 5637 cells was determined using a colony formation assay. T24 and 5637 cells were plated in 6-well plates at a density of 100 cells per well and cultured for 12 h. Thereafter, the cells were treated with metformin at 5, 10, 15, and 20 mmol/L, or with medium only (RPMI-1640 medium + 10% FBS). The culture medium was changed every 3 days and observed continuously for at least 10–12 days. Thereafter, 4% paraformaldehyde was used to fix the cells, followed by staining with using 0.1% Crystal violet solution. The stained cells were counted under an inverted microscope (Olympus).

### Assay of cell apoptosis

Cell apoptosis was detected using flow cytometry. Logarithmic growth phase cells were digested with 0.25% trypsin, and a single cell suspension was prepared. The density of the cells was adjusted to 1.0 × 10^5^/mL, and the cells were inoculated in a 6-well plate at 1 mL/ well. The cells were cultured in an incubator for 24 h. After cell adherence, we discarded the medium and the cells were divided into the control group and the metformin group, which was treated with 5, 10, 15, and 20 mmol/L metformin for 48 h, respectively, and the control group was treated with the same volume of medium. Cells in each group were collected and rinsed with precooled PBS three times. After centrifugation at 1500×*g* for 5 min at 4 °C, the supernatant was discarded, the cells were resuspend in AnnexinV-FITC binding solution, mixed with PI staining solution, and incubated in the dark for 15 min. The apoptosis rate was detected by flow cytometry, carried out according to the manufacturers’ protocol (Cell Sorter BD FACSAria II, BD Biosciences).

### Western blotting analysis

Logarithmic phase cells were and digested using 0.25% trypsin to prepare a single cell suspension, and the cell density was adjusted to 1.0 × 10^6^/mL. The cells were inoculated in a 6-well plate at 1 mL/well, and cultured in an incubator for 24 h. After the cells had attached to the wall, we discarded the medium and the cells were divided into control group and metformin group. The metformin group was treated with 5, 10, 15, and 20 mmol/L metformin for 48 h, and the control group was treated with the same volume of medium containing 10% FBS. The cells were collected, cell lysis buffer containing protease and phosphatase inhibitors was added, and the cells were lysed on ice for 20 min. After centrifugation at 1500 × *g* for 5 min at 4 °C, the supernatant was retained and its protein concentration was determined using the bicinchoninic acid (BCA) method. Then, 40 μg of protein was subjected to SDS–polyacrylamide gel electrophoresis, and the separated proteins were transferred to 0.45 μm or 0.22 μm polyvinylidene fluoride (PVDF) membranes, and blocked using 5% skimmed milk at room temperature for 40 min. Primary antibodies were added (the dilution ratio of cleaved-caspase 3 and cleaved-PARP was 1:500, the dilution ratio of PI3K, p-PI3K, Akt, p-Akt, mTOR and p-mTOR was 1:1000, and the dilution ratio of GAPDH was 1:10,000), and placed in a refrigerator at 4 °C for overnight incubation. The membranes were washed with 1 × Tris-buffered saline-Tween 20 (TBST) three times for 5 min each time and incubated with labeled secondary antibody at room temperature for 40 min. After washing with 1 × TBST three times (5 min each time), ECL exposure luminescence solution was added at a 1:1 ratio onto the PVDF membrane for luminescence development. The immunoreactive protein bands were analyzed using Image-J software (NIH, Bethesda, MD, USA). The relative level of each protein was represented by the ratio of the gray level of target protein band to the gray level of internal reference, GAPDH. For the phosphorylated proteins, the relative expression level was represented by the ratio of the gray level of the phosphorylated target protein to that of the total target protein. Finally, the ChemiDocxRS imaging system was used to obtain images of the protein bands, which were analyzed using Quantity One software (Bio-Rad).

### Statistical analysis

SPSS 22.0 statistical software (IBM Corp., Armonk, NY, USA) was used for the statistical analyses. Measurement data were expressed as the mean ± SD. One-way analysis of variance was used for comparisons between multiple groups. GraphPad Prism 8.0 (GraphPad inc., La Jolla, CA, USA) was used to draw the graphs. Image-J and Quantity One software were used to calculate the western blotting gray values, cell counts, cell clones, and wound areas. Differences were judged to be statistically significant at a *P* value < 0.05 (Additional file 1).

## Results

### Different concentrations of metformin inhibited the migration of T24 and 5637 cells in vitro

**Fig. 1:**
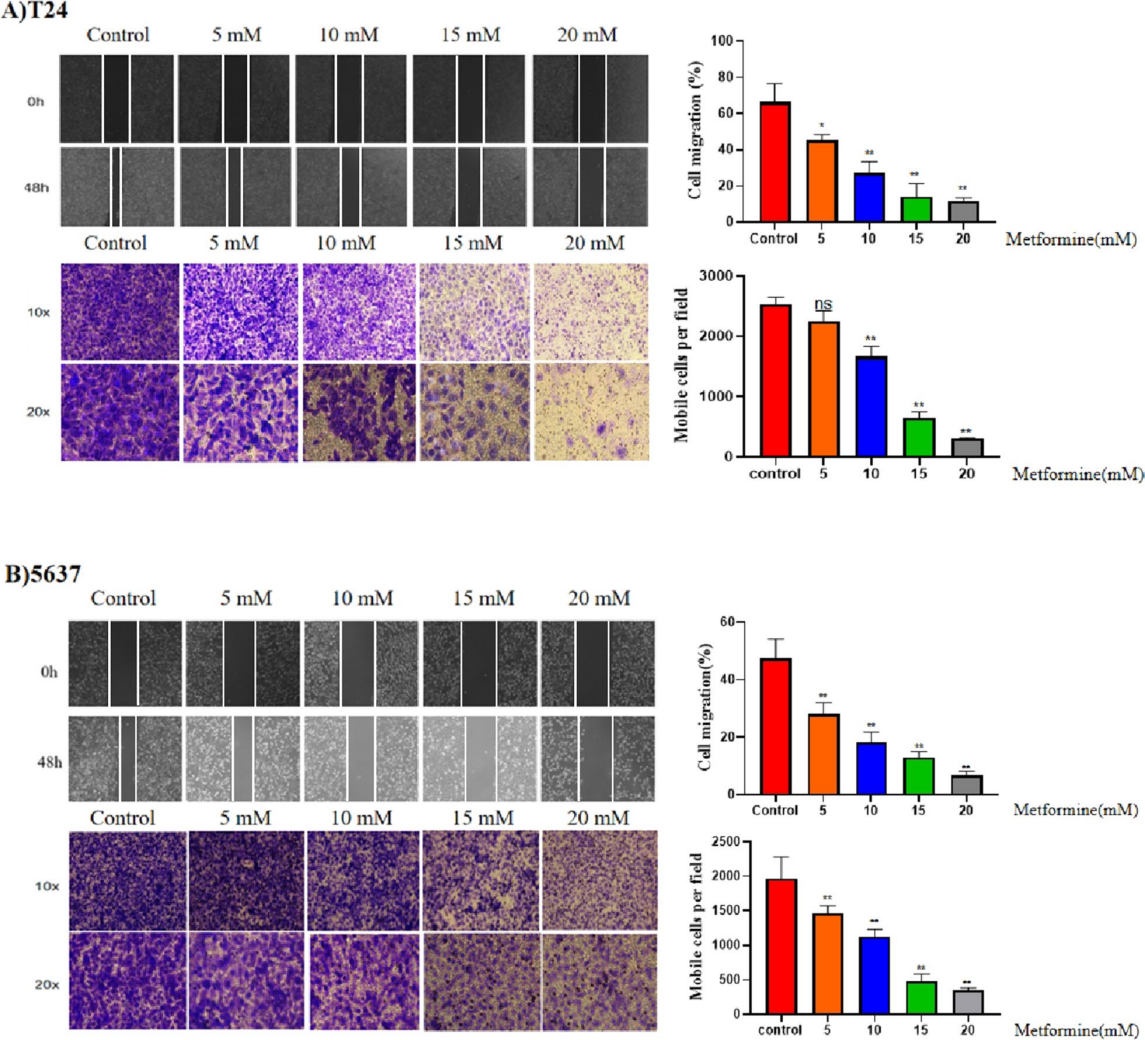
The effect of metformin on the migration of T24 and 5637 cell lines. The effect of different concentrations of metformin on the migration ability of T24 (**A**) and 5637 (**B**) cell lines was observed for 0–48 h (magnification, 10–20 ×). The results are shown as the mean ± the standard deviation (SD) from three independent experiments and were compared with the values for the blank control (*P < 0.05, **P < 0.01) (Original wound healing assay are presented in Additional file 1: Figure 1A B)

Metformin at different concentration inhibited the migration of T24 and 5637 cells, as demonstrated by wound healing assay and Transwell chamber assays (Fig. 1A,B). The migration ability of T24 and 5637 cells decreased with increasing metformin concentration and the higher concentration of metformin inhibited the migration of T24 and 5637 significantly compared with that of the control (*P* < 0.05).

### T24 and T5637 cell proliferation was inhibited by different concentrations of metformin in vitro

**Fig. 2:**
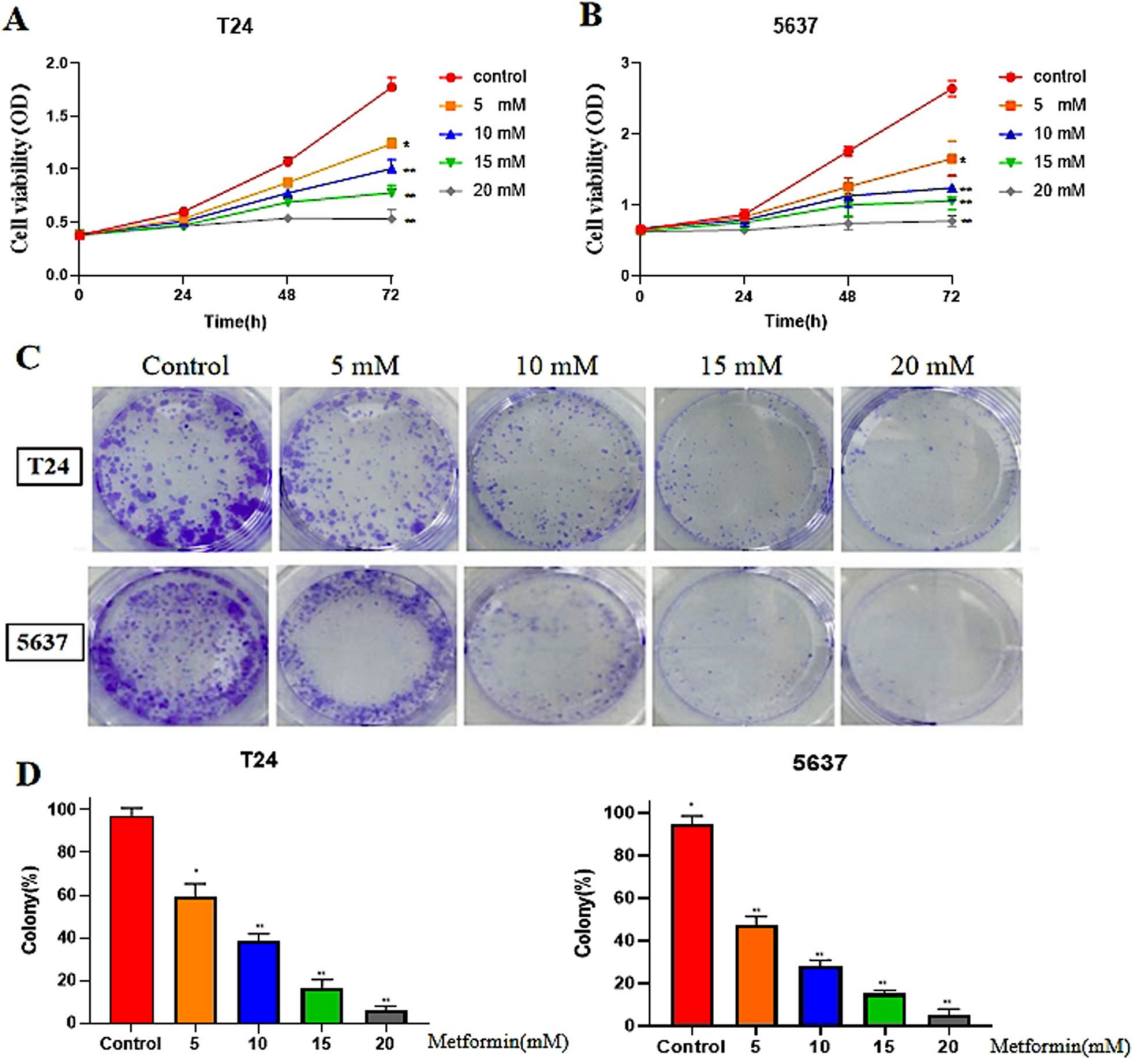
The effects of metformin at different concentrations on the proliferation of T24 and 5637 cell lines. **A** The effects of different concentrations of metformin over time on the proliferative ability of T24 cell lines were observed using CCK-8 assays to measure proliferation. **B** The effects of different concentrations of metformin over time on the proliferative ability of 5637 cell lines were observed using CCK-8 assays to measure cell viability. **C**, **D** The effects of metformin at different concentrations on the proliferation of T24 and 5637 cell lines assessed using colony formation experiments. The cell clones were counted and the clone formation rate of each drug concentration treatment group was calculated using Quantity One software. The results are shown as the mean ± the standard deviation (SD) from three independent experiments and were compared with the values for the control (*P < 0.05, **P < 0.01). CCK-8, cell counting kit 8

The effects of different concentrations of metformin on T24 and 5637 cell proliferation were tested using CCK-8 assays (Fig. 2A,B) and colony formation assays (Fig. 2C,D). The proliferative ability of T24 and 5637 cells was inhibited by increasing concentrations of metformin compared with the control (*P* < 0.05).

### Different concentrations of metformin promoted apoptosis of T24 and T5637 cells in vitro

**Fig. 3:**
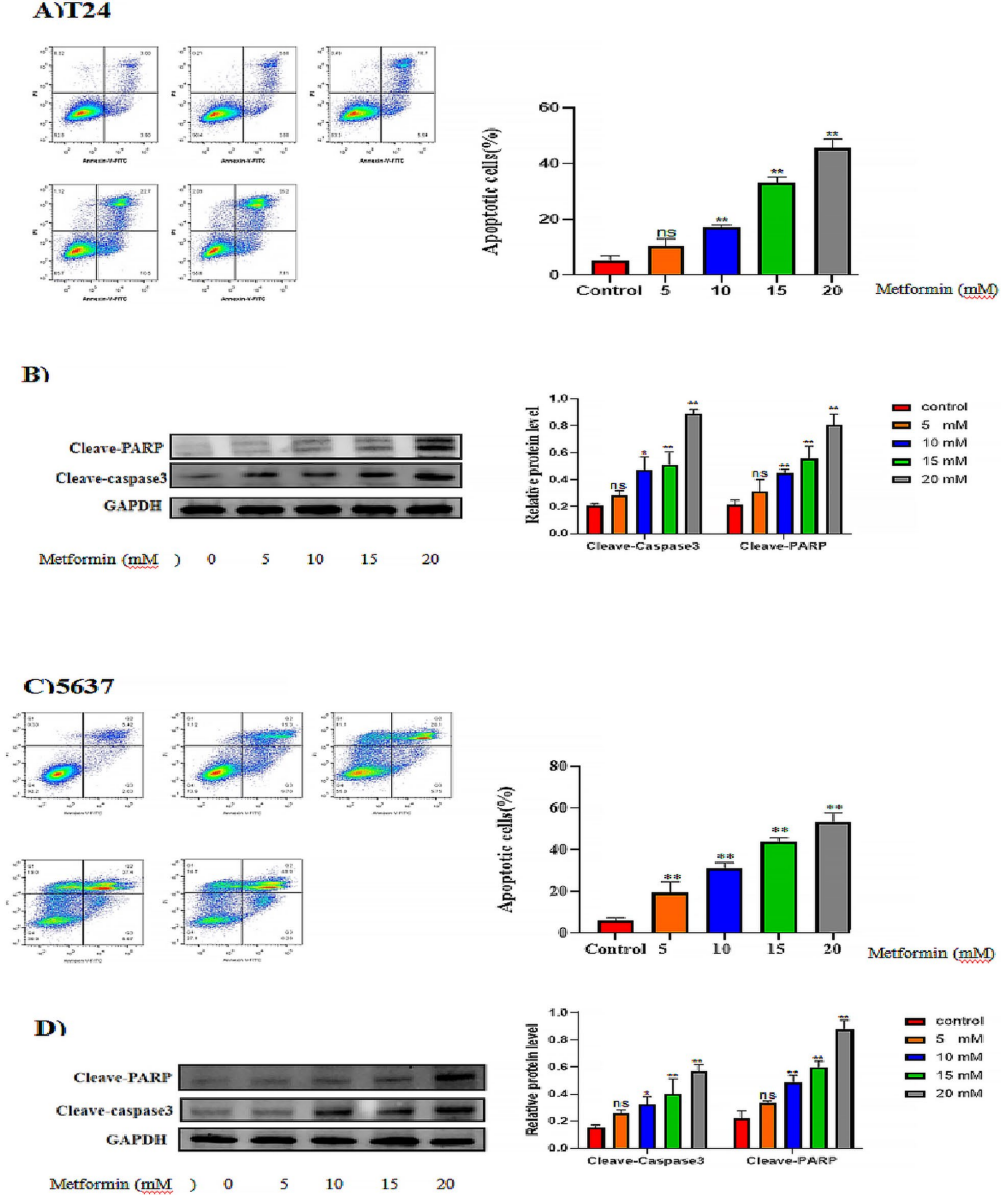
In vitro, cell apoptosis was promoted by different of concentrations of metformin. **A** T24 cell apoptosis as detected using flow cytometry. The images show the number of Q2 + Q4 as apoptotic cells. **B** Western blotting detection of the levels of cleaved-caspase 3 and cleave-PARP in T24 cells. Full length blots/gels are presented in Additional file 1: Figure 3B. The loading control comprised GAPDH. Full length blots/gels are presented in Additional file 1: Figure 3B, 4A. **C** 5637 cell apoptosis as detected using flow cytometry. The images show the number of Q2 + Q4 as apoptotic cells. **D** Western blotting detection of the levels of cleaved-caspase 3 and cleave-PARP in 5637 cells. Full length blots/gels are presented in Additional file 1: Figure 3D. The loading control comprised GAPDH. Full length blots/gels are presented in Additional file 1: Figure 3D, 4B. The representative column diagrams showing results of relative protein expression. In the graphs, the results are shown as the mean ± the standard deviation (SD) from three independent experiments and were compared with the values for the control (*P < 0.05, **P < 0.01). PARP, poly(ADP-ribose) polymerase GAPDH, glyceraldehyde-3-phosphate dehydrogenase

Metformin increased the rate of apoptosis in a concentration-dependent manner compared with that of the control (*P* < 0.05; Fig. 3). The highest concentration of metformin significantly promoted cell apoptosis compared with that of the control (*P* < 0.05). The levels of cleaved-caspase-3 and cleaved-PARP increased significantly in T24 and 5637 cells treated with highest concentration of metformin (*P* < 0.05). Interestingly, the lowest concentration of metformin did not increase the level of cleaved-caspase 3 and cleaved-PARP (*P* > 0.05), but with the increase in metformin concentration, cleaved-caspase 3 and cleaved-PARP protein levels increased significantly (*P* < 0.05).

### The PI3K/AKT/mTOR pathway is inhibited by different concentrations of metformin

**Fig. 4:**
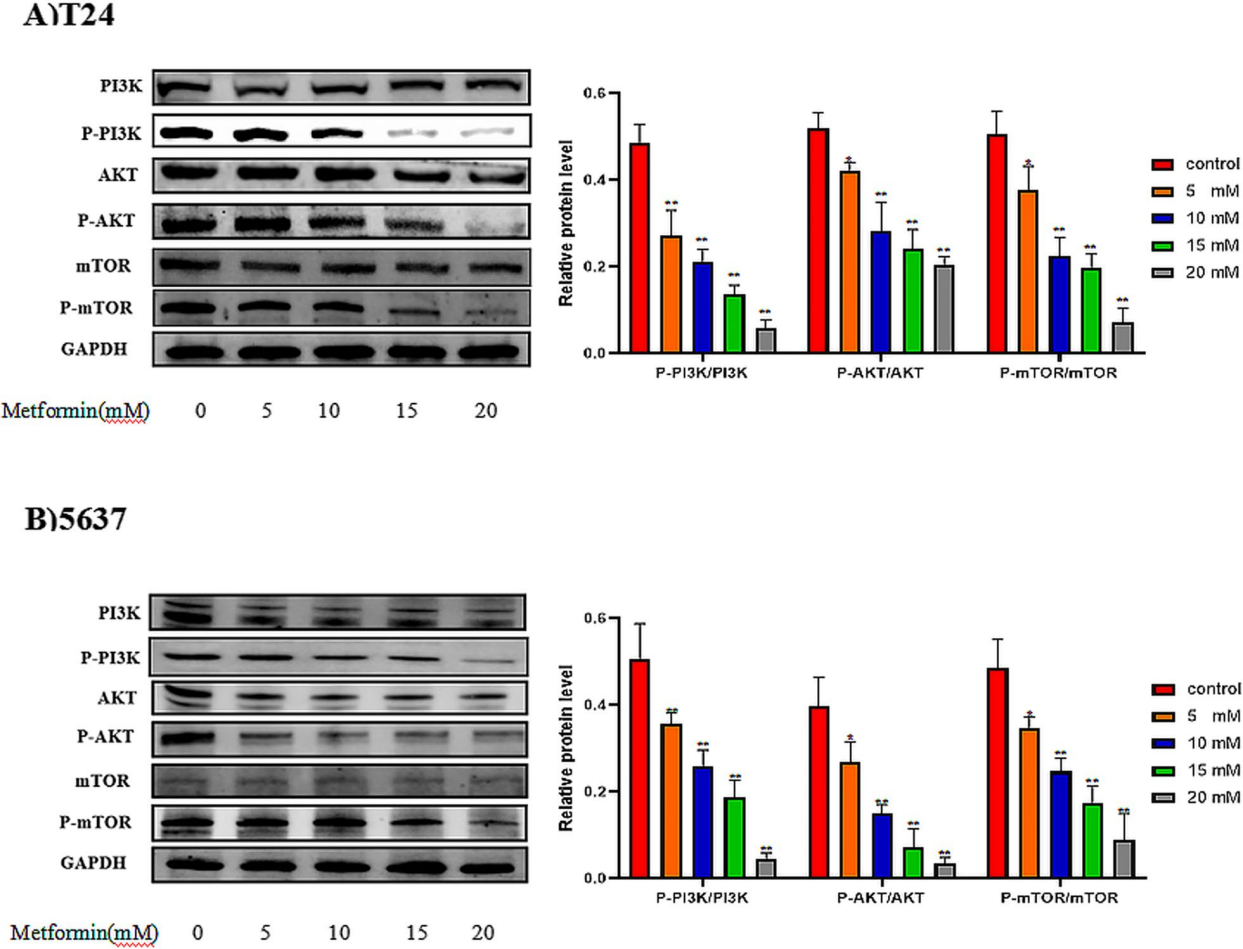
The different of concentrations of Metformin inhibited PI3K/AKT/mTOR pathway activation in vitro. **A** Western blotting detection of PI3K/AKT/mTOR and p-PI3K/p-AKT/p-mTOR protein levels in T24 cells. Full length blots/gels are presented in Additional file 1: Figure 4A. The loading control comprised GAPDH.  Full length blots/gels are presented in Additional file 1: Figure 3B, 4A. The graph shows the relative protein levels. **B** Western blotting detection of PI3K/AKT/mTOR and p-PI3K/p-AKT/p-mTOR protein levels in 5637 cells.  Full length blots/gels are presented in Additional file 1: Figure 4B. The loading control comprised GAPDH. Full length blots/gels are presented in Additional file 1: Figure 3D, 4B. The graph shows the relative protein levels. Data are shown as the mean ± SD of three independently performed experiments (*P < 0.05, **P < 0.01). PI3K, phosphatidylinositol-4,5-bisphosphate 3-kinase; p-PI3K, phosphorylated PI3K; Akt, protein kinase B; p-Akt, phosphorylated Akt; mTOR, mechanistic target of rapamycin; p-mTOR, phosphorylated mTOR; GAPDH, glyceraldehyde-3-phosphate dehydrogenase

PI3K, Akt, and mTOR phosphorylation in T24 and 5637 cells was significantly reduced by metformin in a concentration-dependent manner (*P* < 0.05; Fig. 4).

## Discussion

In the urinary system, the most common malignancy is bladder cancer, ranking as the 4th most common malignancy in men and the 11th most common malignancy in women in the United States. The incidence and mortality of bladder cancer in males is approximately four times that of females [1]. In China in 2019, there were 549,393 cases of bladder cancer, with an initial incidence of 7.2 per 100,000, ranking as 10th among all tumors. There were almost the same number of new cases in China (82,270) as there were in the in the USA (82,501) [2]. Clinically, about 70% of bladder cancers are NMIBC and 30% are MIBC. NMIBC has a low mortality rate and a high recurrence rate, whereas about 50% of cases of MIBC are potentially fatal [3]. For patients with NMIBC, the preferred treatment is TURBT with conventional postoperative infusion chemotherapy using the Bacillus Calmette–Guérin (BCG) vaccine (BCG), epirubicin (EPI), pirubicin (THP), and other cytotoxic drugs to prevent postoperative tumor recurrence and progression [4]. Despite active postoperative adjuvant treatment, the 5-year recurrence rate remains high (50–70%) among patients [5, 6].

Metformin safely reduces insulin resistance and lowers blood glucose [7, 8]. It is commonly used in the clinic to treat type II diabetes mellitus and has become popular in tumor treatment research because of its extensive anti-tumor effects. Metformin has anti-tumor effects in a variety of tumors, such as breast cancer, colon cancer, stomach cancer, pancreatic cancer, prostate cancer, lung cancer, and liver cancer [9–14]. Our current understanding of the mechanism by which metformin inhibits tumor cell invasion, migration, and proliferation can be roughly divided into the following pathways [9–15]: (1) Metformin inhibits the downstream mTOR pathway by activating the AMP-activated protein kinase (AMPK) signaling pathway to inhibit tumors; (2) Metformin inhibits tumors by activating reactive oxygen species (ROS) and the C-Jun N-Terminal Kinase 1 (JNK) signaling cascade; (3) Metformin can enhance the cytotoxicity of chemotherapy drugs. However, there has been only one study on the molecular mechanism of metformin when used in bladder cancer [16]. Our results indicated that under 5, 10, 15, and 20 mmol/L metformin treatment for 48 h, T24 and 5637 cell survival was lower compared with that of the control group. The migration ability of T24 and T5637 cells treated with metformin was significantly weakened in a concentration-dependent manner, as assessed using wound healing and Transwell chamber assays. Thus, metformin can inhibit the migration and proliferation abilities of T24 and T5637 cells, which suggested that it would inhibit bladder cancer progression.

Programmed cell death (PCD) comprises mainly apoptosis (type I) and autophagy (type II) (17). The most common type of PCD is apoptosis, which is under the control of both intracellular and extracellular signals. Apoptosis is characterized by changes in cell morphology, e.g., mitosis and DNA condensation, production of membranous vesicles, cell contraction, and the development of apoptotic bodies. In last 30 years, apoptotic signaling pathways have been intensively studied in many types of tumor cells. Tumor apoptosis-related protein include the caspase family and the B-cell CLL/lymphoma 2 (Bcl-2) family, that latter of which has vital functions mitochondria-mediated apoptosis. The pro-apoptotic BCL2 associated X protein (Bax) promotes mitochondrial cytochrome C through regulating the permeability of the mitochondrial membrane and activating the caspase cascade reaction, which ultimately leads to cell apoptosis. In our apoptosis experiment, the level of the apoptosis promoter cleaved-caspase 3 was increased by the different concentrations of metformin. In addition, flow cytometry showed that a higher proportion of T24 and T5637 bladder cancer cells underwent apoptosis after treatment with different concentrations of metformin.

Tumorigenesis and development are complex processes and extensive research on the molecular mechanism of tumors has revealed that tumorigenesis and development are related to multiple signaling pathways, among which PI3K/Akt /mTOR is the canonical pathway. Domestic and foreign scholars have discovered that this signaling pathway plays a crucial role in the occurrence and development of breast cancer, cervical cancer, pancreatic cancer, and other tumors [17]. In ovarian cancer, Parashar established MCW-OV-SL-3, an endometrioid subtype of ovarian cancer cell line, which is tumorigenic and highly metastatic, similar to the A-2780 ovarian cancer cell line. They observed that aberrant PI3K/Akt/ERK signaling promotes cancer stemness characteristics, chemoresistance, and EMT in this cell line. Meanwhile, inhibition of PI3K/Akt/ERK signaling using a PI3K/Akt dual kinase inhibitor abolished oncogenic features such as cancer stemness, chemoresistance, and EMT [18]. In breast cancer, the PI3K pathway presents mutations of genes that encode the catalytic and the regulatory subunits. The most frequent mutations are in *PIK3CA*, expecially in exon 9 and 20, identified from tumor tissue and/or circulating DNA, in all breast cancer subtypes. Breast cancer mutations also appear in receptor tyrosine kinase receptors, such as HER2, and phosphorylation of this receptor leads to PI3K/Akt/mTOR activation. PI3K mutations, PTEN methylation, and Akt activation will result in hormonal therapy resistance [19]. In colon cancer, metformin monotherapy was more effective than active vitamin D3 (VD3) against cancer, resulting in higher levels of p21, p27, phosphatase and tensin homolog (PTEN), BCL2-associated X protein (BAX), cytochrome C (Cyto-C),and caspase-3(Casp-3). Metformin also inhibited colon oncogenesis involving abnormal upregulation of the cyclin D1 (CCND1), CCND3, B-cell lymphoma 2 (BCL2), and PI3K/Akt/mTOR networks; and induced higher rates of apoptosis, both in vivo and in vitro. Although all dual regimens showed enhanced regulation of the PI3K/PTEN/Akt/mTOR pathway, together with higher expression of cell cycle inhibitors and pro-apoptotic molecules compared with monotherapy, the Metformin/5-fluorouracil combination was better than the other dual therapies. In contrast, triple therapy regimens exhibited the best anticancer effects associated with cell cycle arrest and apoptosis compared with single and dual regimens, possibly through enhanced attenuation of the PI3K/Akt/mTOR oncogenic pathway [20]. In summary, PI3K/Akt signaling is associated with cell autophagy, apoptosis, proliferation, and inflammation. In-depth study of the PI3K signaling pathway has important clinical significance for tumor therapy. In addition, in this pathway, mTOR acts a downstream regulator, exerting a vital function in autophagy and protein synthesis [21–24]. Although there have been few studies about metformin in bladder cancer, analysis of reviews and published studies revealed that more than 40% of patients with urothelial carcinoma had a dysregulated PI3K/AKT/mTOR pathway [25–30]. These observations inspired us to consider whether metformin also has an inhibitory effect in bladder cancer and if this effect is related to the PI3K pathway. In our in vitro study, we found that metformin indeed inhibited the PI3K/Akt/mTOR signaling pathway, and different concentrations of metformin reduced the levels of phosphorylated PI3K, Akt, and mTOR.

In conclusion, metformin inhibits bladder cancer T24 and 5637 cell migration and proliferation, activates the caspase cascade signaling pathway, and induces cell apoptosis. The mechanism might be associated with inhibition of the activation of the PI3K/Akt/mTOR signaling pathway.

## Supplementary Information

**Additional file 1.** Supplementary figures.

## Data Availability

The datasets used and/or analyzed during the current study are available from the corresponding author or the first author on reasonable request.

## Abbreviations

BC: Bladder cancer
PI3K: Phosphatidylinositol-4,5-bisphosphate 3-kinase
p-PI3K: Phosphorylated (p)-PI3K
Akt: Protein kinase B
p-Akt: Phosphorylated Akt
mTOR: Mechanistic target of rapamycin
p-mTOR: Phosphorylated mTOR
GAPDH: Glyceraldehyde-3-phosphate dehydrogenase
PARO: Poly(ADP-ribose) polymerase (PARP)
