# Supplementary material for: Metformin exerts an antitumor effect by inhibiting bladder cancer cell migration and growth, and promoting apoptosis through the PI3K/AKT/mTOR pathway
Source: BMC Urol. 2022 May 24;22:79. doi: 10.1186/s12894-022-01027-2 (PMC9131696; doi:10.1186/s12894-022-01027-2)

Figure 1A.wound healing assay

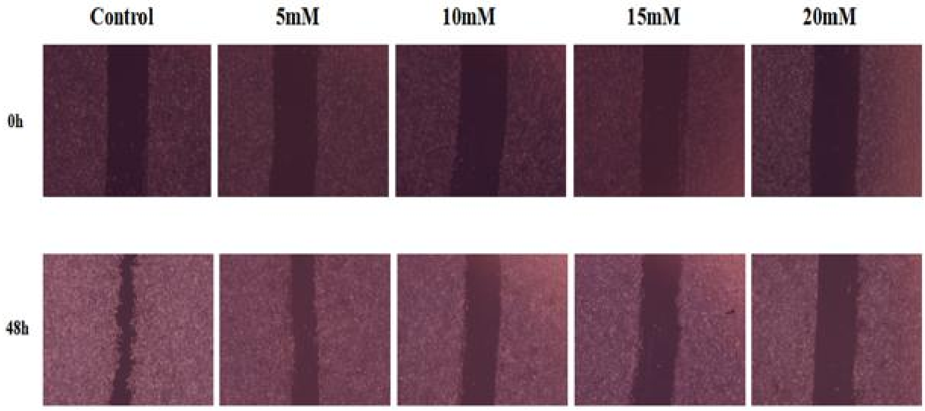

Figure 1B.wound healing assay

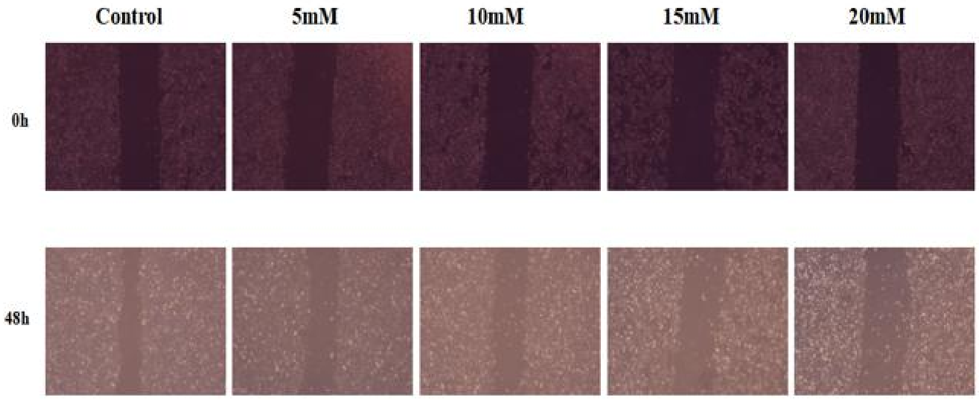

Figure 3B.Cleave-parp（24Kda）

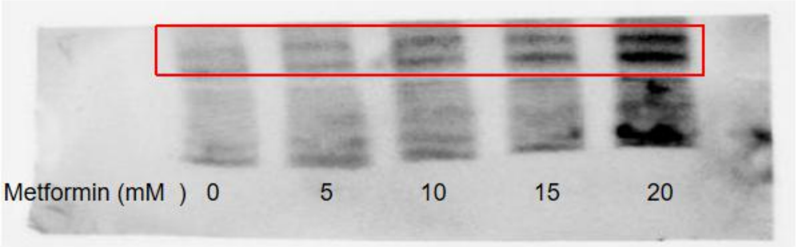

Figure 3B.Cleave-Caspase3（17Kda）

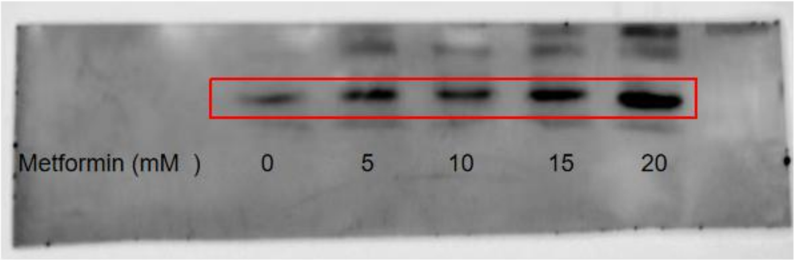

Figure 3B,4A. GAPDH（36Kda）

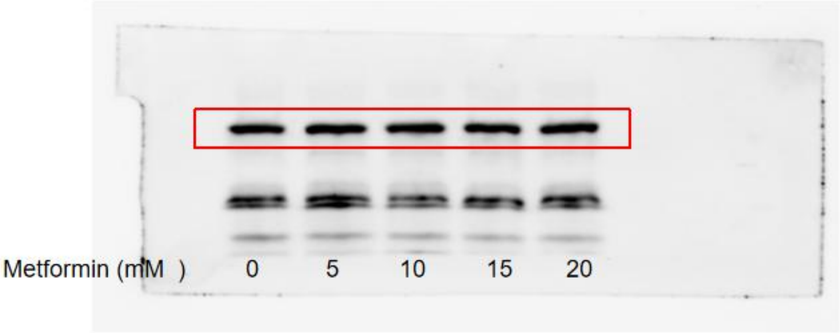

Figure 3D.Cleave-parp（24Kda）

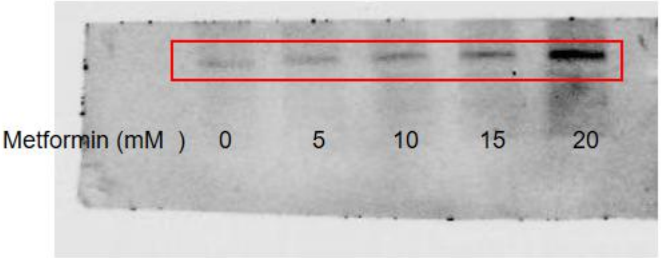

Figure 3D.Cleave-Caspase3（17Kda）

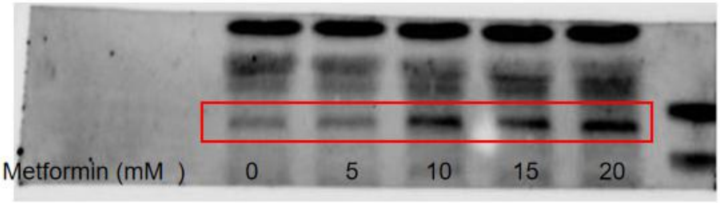

Figure 3D,4B.   GAPDH（36Kda）

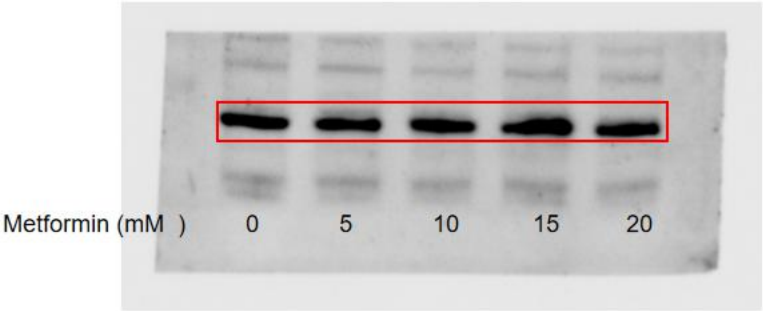

Figure 4A. PI3K（85Kda）

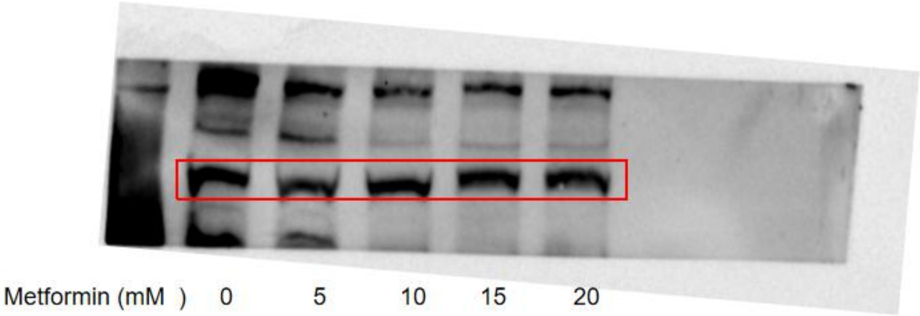

Figure 4A. P-PI3K（85Kda）

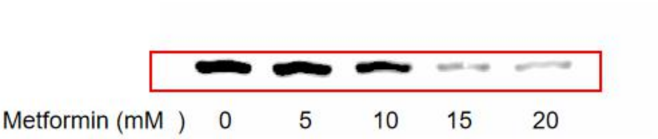

Figure 4A. AKT（60Kda）

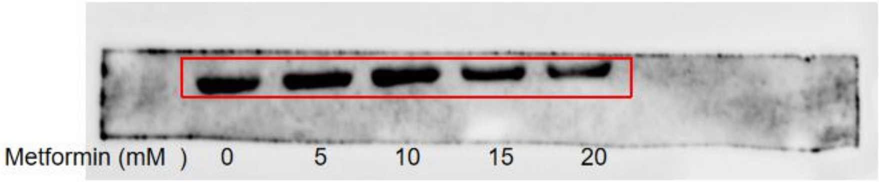

Figure 4A. P-AKT（60Kda）

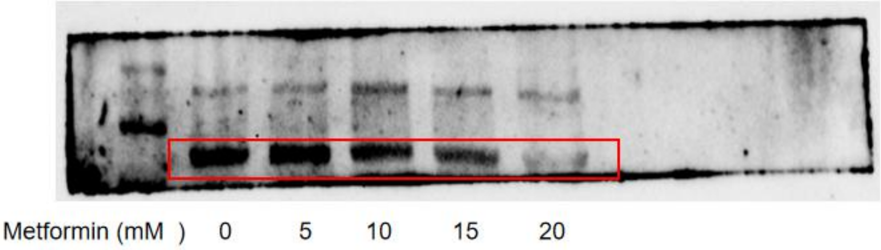

Figure 4A. mTOR（289Kda）

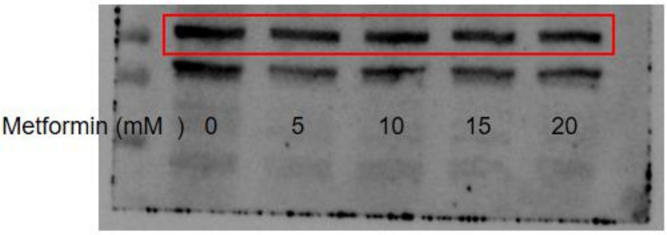

Figure 4A. P-mTOR（289Kda）

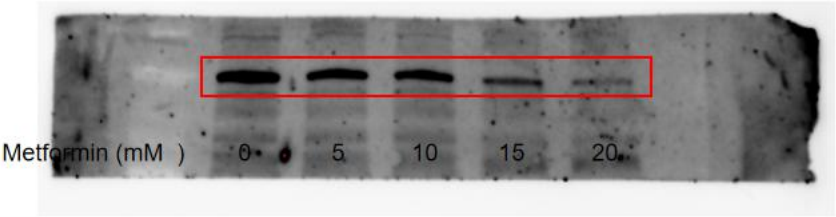

Figure 4B. PI3K（85Kda）

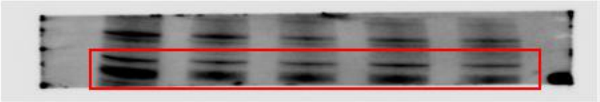

Metformin (mM) 0 5 10 15 20

Figure 4B. P-PI3K（85Kda）

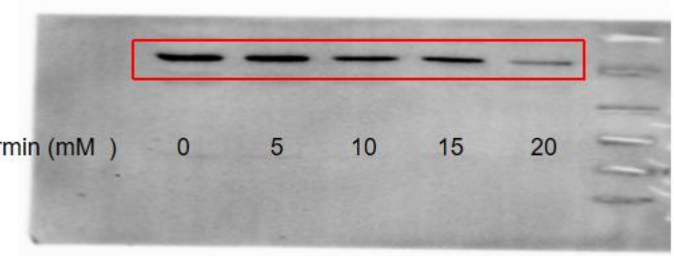

Figure 4B. AKT（60Kda）

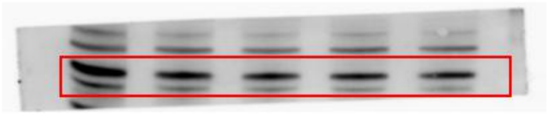

Metformin (mM) 0 5 10 15 20

Figure 4B. P-AKT（60Kda）

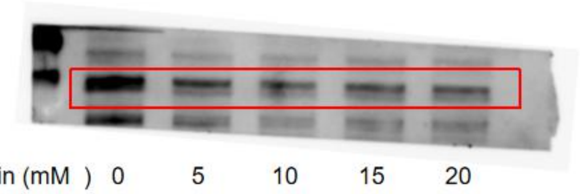

Metformin (mM) 0 5 10 15 20

Figure 4B. mTOR（289Kda）

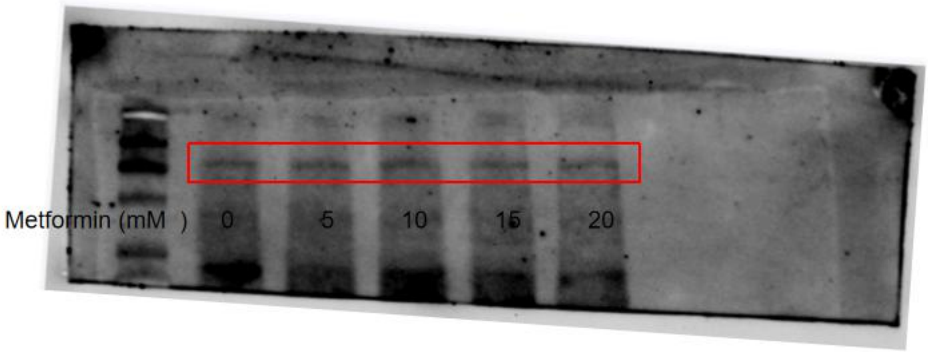

Figure 4B. P-mTOR（289Kda）

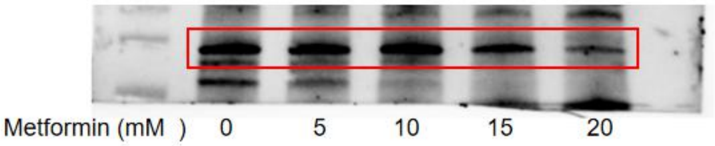

cBIOPORTAL software results

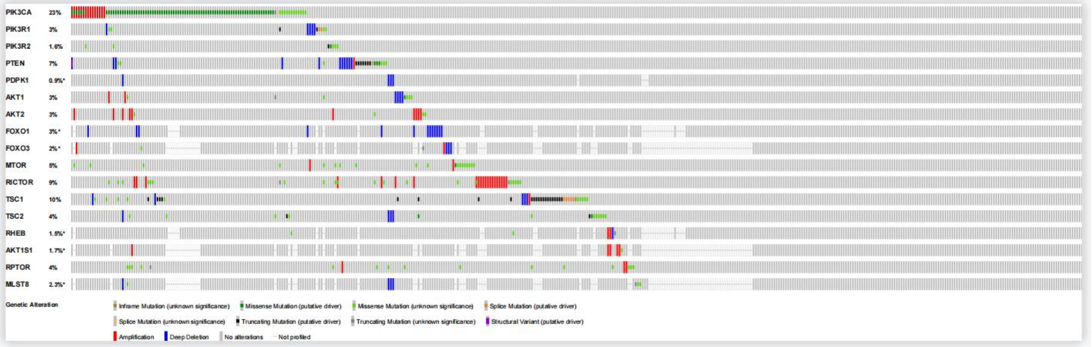

Supplement: Supplementary file 1 — Additional file 1. Supplementary figures. [file 12894_2022_1027_MOESM1_ESM.pdf]
